# Host Protective Mechanisms to Intestinal Amebiasis

**DOI:** 10.1016/j.pt.2020.09.015

**Published:** 2020-10-23

**Authors:** Md Jashim Uddin, Jhansi L. Leslie, William A. Petri

**Affiliations:** 1 Division of Infectious Diseases and International Health, Department of Medicine, University of Virginia, Charlottesville, VA, USA

## Abstract

The protozoan parasite *Entamoeba histolytica* is the causative agent of amebiasis, an infection that manifests as colitis and, in some cases, liver abscess. A better understanding of host protective factors is key to developing an effective remedy. Recently, significant advances have been made in understanding the mechanisms of MUC2 production by goblet cells upon amebic infection, regulation of antimicrobial peptide production by Paneth cells, the interaction of commensal microbiota with immune stimulation, and host genetics in conferring protection from amebiasis. In addition to host pathways that may serve as potential therapeutic targets, significant progress has also been made with respect to development of a vaccine against amebiasis. Here, we aim to highlight the current understanding and knowledge gaps critically.

## Outline of Amebiasis

*Entamoeba histolytica* infection accounts for millions of symptomatic infections and about 55 000 deaths worldwide annually [1]. Infection occurs when **amebic cysts** (see Glossary) enter the host intestinal lumen via contaminated food and water. In the lumen, the cyst produces trophozoites which can invade intestinal epithelial cells. Trophozoites adhere to intestinal epithelial cells by the parasite's surface **Gal/GalNAc lectin** which binds to host cell membrane carbohydrates galactose (Gal) and/or *N*-acetyl-d-galactosamine (GalNAc) [2]. After adhering to host cells, an ameba uses several cytotoxic mechanisms to induce cell killing and tissue invasion, including apoptosis, phagocytosis, and trogocytosis [3,4] (Box 1).

Besides the symptomatic cases, a large number of infections remain asymptomatic [5]. Host defense mechanisms presumably contribute to these asymptomatic infections. In recent years, significant advancement has been made in our understanding of the mechanisms through which the MUC2 mucin protects from amebic infection [6-8]. Epithelial cells, in addition to mucin production, can also promote protection by producing **antimicrobial peptides** (**AMPs**) and cytokines [9-11]. In addition to the host epithelium, the role of the microbiome has re-emerged as important in determining protection from amebic infection [12-15]. Our group's recent work has built a framework for how different members of the bacterial microbiota can communicate with bone marrow progenitor cells, resulting in amebic protection [16]. We also explored the impact of cell-mediated immunity in amebiasis [12,17]. Building on our early findings – that the amebic surface antigen LecA is critical for colonization – work over the last couple of decades has focused on developing an effective vaccine against amebiasis. By adding liposome-based **adjuvants** to LecA, the efficacy of the vaccine has been recently improved [18,19]. Here, we outline the recent progress on host response to amebiasis.

## The Host Mucus Layer Plays the Role of Primary Sentinel against *E. histolytica* Infections

The mucus layer keeps the approximate 100 trillion microorganisms away from the intestinal epithelial layer and confers the first line of defense in the gut against harmful infections. MUC2 mucin is the primary component of the mucus layer and is known to be essential for the protection from detrimental intestinal insults [20]. MUC2 mucin was shown to be involved in the maintenance of a healthy microbiota, with transplantation of *Muc2*^−^/^−^ microbiota making *Muc2*^+^/^+^ mice more susceptible to dextran sodium sulfate colitis [21].

The protective role of the host's mucin layer against *E. histolytica* is well studied. Chadee *et al*. isolated and purified colonic mucus from rat and human colon and showed that even the crude mucus could inhibit amebic adherence to Chinese hamster ovary (CHO) cells by up to 70% and prevented killing of rat colonic epithelial cells by amebic trophozoites by up to 40% [22]. *In vivo* studies using closed colonic loops in *Muc2*^−^/^−^ mice revealed severe colonic disease and a substantially higher expression of inflammatory cytokines compared with wild-type (WT) mice [23]. It will be important to follow up these studies. While the colonic loop model has been useful in unraveling host–parasite interactions, it does not mimic the natural infection model in several ways. In this model, infection is forced to be confined in a certain portion of the colon. Also, mice are harvested after only several hours of amebic challenge, which does not allow long-term colonization. To understand the role of MUC2 in long-term colonization with ameba, a different model with several days of established infection is required (Box 2).

*E. histolytica* needs to cleave the mucus surface and attach to intestinal epithelial cells to establish an infection. Although several **cysteine proteases**, including EhCP1, EhCP2, and EhCP5, are expressed by *E. histolytica*, only the role of EhCP5 in amebiasis is well established. *In vitro* studies have revealed that the C-terminal cysteine-rich domain of MUC2 is cleaved off by EhCP5 [6,24]. Since MUC2 is a major component of the mucin layer, EhCP5 facilitates mucin degradation by cleaving it, which allows *E. histolytica* to overcome the mucin barrier. EhCP5-deficient *E. histolytica* trophozoites were unable to cleave the mucus layer in LS174T epithelial cells derived from human colon; however, trophozoites were able to kill the cells without the mucus layer, suggesting that cysteine proteases are critical for cleaving the mucins but not for amebic cytotoxicity [25].

Once trophozoites pass the mucin layer, and attach to the intestinal epithelial cells, there is an immediate stimulation of mucin production by the goblet cells to impose a secondary mucin shield. The **pathogen-associated molecular patterns** (**PAMPs**) and the mechanism of this attachment-induced mucin production were not quite understood until recently. Cornick *et al*. discovered that induction of mucin secretion by goblet cells is mostly mediated by the interaction of EhCP5 with goblet cell αvβ3 integrin. This interaction activated SRC family kinases, which, in turn, phosphorylated and activated phosphatidylinositol-3-kinase (PI3K). PI3K activation resulted in the activation of protein kinase (PK)Cδ to drive vesicle-mediated exocytosis of mucin [6]. Using monolayers of the mucin-producing LS174T cell line, the authors found that challenge with WT trophozoites resulted in a fivefold increase in mucin, while EhCP5-silenced trophozoites increased mucin production by only twofold. While it is clear that EhCP5 plays a major role in mucus secretion, these results suggest that other important players have yet to be investigated. Vesicle-mediated mucin exocytosis is critical from protection from amebic damage. Knocking down of vesicle-associated membrane protein 8 (VAMP8) in LS174T cells inhibited mucin generation and facilitated the attachment and cell killing by trophozoites. As expected, *VAMP*^−/−^ mice had more colonic pathology and inflammation compared with littermate controls [8]. Interestingly, there were noticeably higher MUC2-positive goblet cells in *VAMP*^−/−^ mice compared with *VAMP8*^+/+^ mice, suggestive of inefficient mucin exocytosis but not mucin production in *VAMP8*^−/−^ mice.

In addition to MUC2, there are other mucins that are important for protection from colitis. One important cell surface mucin is MUC1. *Muc1* expression was observed to be increased in the small intestine, cecum, and colon of mice upon infection with the bacterial pathogen *Campylobacter jejuni* [26]. In support of the importance of MUC1 in protection, patients with ulcerative colitis had increased expression of *Muc1* in inflamed tissue [27]. Expression of another secretory mucin, MUC6, was observed to be increased in amebic infected colonic tissue [23]. These data suggest that other mucins, in addition to MUC2, may play a role in protection from amebiasis. These areas are open to being investigated to understand the complex interaction between host and *E. histolytica* in the context of mucus response.

## Trophozoites That Pass the Mucus Layer Experience Secondary Host Defense at the Epithelial Layer

In response to attachment of the parasite, different functional and regulatory changes occur in epithelial cells. A recent study showed that EhCP112 is involved in degrading tight-junction proteins and reducing transepithelial electrical resistance [28]. One of the first responses is by the goblet cells that produce mucins. In addition to mucin production, goblet cells can regulate Paneth cell morphology and function; it has been reported that, in *Muc2*^−/−^ mice, the localization of Paneth cells is altered and secretion of cathelicidin is decreased, while the secretion of α-defensins is increased [7,29]. Studies have revealed the amebicidal activity of α-defensins and β-defensins. *In vitro* treatment with cryptdin-2 (an α-defensin) damaged the structural integrity of *E. histolytica* trophozoites and diminished the synthesis of DNA, RNA, and protein [11]. Expression of human β-defensin-2 (HBD-2) mRNA was shown to be increased in a human colonic cell line (Caco-2) upon exposure to *E. histolytica*. Interestingly, released HBD-2 damaged the membrane integrity of the parasite [9]. Amebic infection can also induce cathelicidin and cathelicidin-related AMPs in human and murine colonic cells, however, the amebicidal role of these compounds is not established [3]. Another AMP expressed by Paneth cells is the REG (islet of Langerhans regenerating protein) family protein. Peterson *et al*. performed a microarray analysis of human colonic biopsies and observed around an eight- to 10-fold increase in expression of REG1A and REG1B mRNA during acute amebic infection [30]. REG1A is known to be antiapoptotic and is induced in the inflamed tissue in patients with inflammatory bowel disease (IBD) [31]. When colonic epithelial cells from *Reg*^−/−^ and littermate WT control mice were incubated with amebic protein there was higher apoptosis of epithelial cells of *Reg*^−/−^ mice. These data suggest that REG1 potentially protects from amebiasis by resisting the amebic induced apoptosis of epithelial cells, though *in vivo* confirmation is missing. REG4 was recently added to this family and was shown to be predominantly expressed by enteroendocrine cells in human and mice colon and is transcriptionally regulated by the ATF2 transcription factor [32]. In the inflamed tissue of ulcerative colitis patients, *Reg4* mRNA expression was higher and was correlated with increased expression of inflammatory cytokines [32]. Future studies should investigate the role of REG4 in infection-induced colitis-like amebiasis.

**Figure 1. Key Figure:**
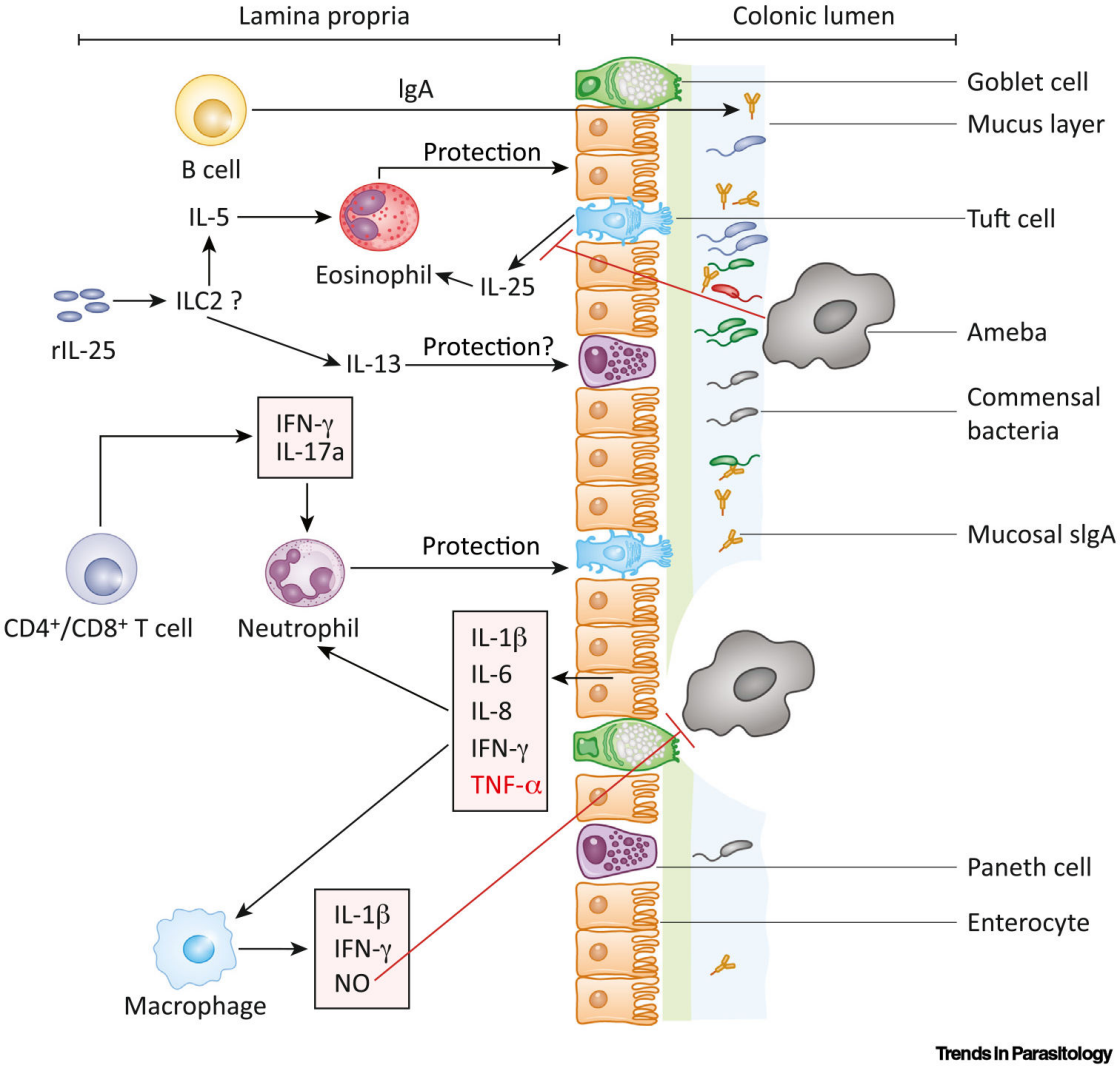
Host Protective Pathways to Amebic Infections For a Figure360 author presentation of Figure 1, see the figure legend at https://doi.org/10.1016/j.pt.2020.09.015. The colonic epithelial layer is covered with a bilayered mucus film. The outer mucus layer contains commensal microbiota and soluble IgA. The mucus layer, combined with commensal microbiota and IgA, acts as the first line of defense against amebic infection. The ameba can cleave off the mucus layer using cysteine proteases and attach to epithelial cells by the surface Gal/GalNAc lectin. Once attached, amebae can damage the epithelial layer and initiate a robust inflammatory response [production of interleukin (IL)-1β, IL-6, IL-8, interferon (IFN)-γ, and tumor necrosis factor (TNF)-α]. The red-colored cytokine TNF-α induces disease progression. Inflammatory cytokines and chemokines recruit immune cells, including neutrophils and macrophages. By releasing reactive oxygen species (ROS), nitric oxide (NO), and inflammatory cytokines, the neutrophils and macrophages can kill the amebae. Intestinal tuft cells are known to be the major source of IL-25. Amebic infection can downregulate IL-25 production. Exogenous recombinant IL-25 treatment can protect through inducing a type 2 immune response followed by the eosinophilia. Exposure to primary infection or LecA can prompt the production of ameba-specific mucosal IgA (by B cells), IFN-γ, and IL-17A (by CD4^+^ and CD8^+^ T cells), which could protect from subsequent amebic infections.

Amebic attachment to epithelium induces the production of proinflammatory cytokines. Using an *ex vivo* model, Bansal *et al*. observed that trophozoites breach the mucus layer within 2 h and stimulate the production of proinflammatory cytokines, including interleukin (IL-)1β, IL-6, IL-8, interferon-gamma (IFN-γ), and tumor necrosis factor-alpha (TNF-α) (Figure 1, Key Figure) [33].

Thus, the epithelium provides at least three layers of protection against amebic colitis: (i) mucins produced by goblet cells deter trophozoite attachment to epithelial cells, (ii) AMPs, secreted primarily from Paneth cells, dampen down the trophozoites functionality and membrane integrity, and (iii) inflammatory cytokines from epithelial cells signal and recruit immune cells to fight parasites that have already passed the barrier. It is likely that tuft cells also play an important role in amebiasis since they produce IL-25, which has been found to be protective [17].

## Host Genetics Plays an Important Role in Determining the Outcome of *E. histolytica* Infection

Studies on Bangladeshi children showed that amebiasis is more common in malnourished children [34]. Leptin, a hormone produced mainly by adipocytes, regulates food intake and energy expenditure and is suppressed in malnourished children. Duggal *et al*. observed that genetic polymorphisms in the leptin receptor were significantly associated with increased *E. histolytica* infection in mice and humans. A strong association was observed for a nonsynonymous **SNP** at the amino acid 223 position of the leptin receptor, with a glutamine (Q) for the WT allele and arginine(R) for the mutant allele [35]. Leveraging the murine model of infection, they found that having RR and QR alleles led to a significantly higher infection rate and the death of intestinal epithelial cells. Leptin signaling in epithelial cells, but not in hematopoietic cells, was critical for protection; however, it was observed that RR mice had impaired recruitment of neutrophils to the site of amebic infection. Neutrophils from polymorphic mice also showed compromised chemotaxis toward leptin *in vitro*, suggesting that leptin-mediated protection might be dependent on neutrophils [36,37]. It is important to mention that activation of signal transducer and activator of transcription (STAT)3, but not SHP-2 and STAT5, was critical for protection mediated by leptin signaling [38].

A **genome-wide association study** was conducted in two birth cohort studies in Bangladesh to identify genetic variations associated with symptomatic *E. histolytica* infection [39]. An association with SNPs in a locus that included *CREM* (cAMP-responsive element modulator) with *E. histolytica* diarrhea was identified [39]. Interestingly, using GTEx data, the polymorphisms were associated with decreased *CREM* expression. In support of this human data, *CREM*-deficient mice were found to be more susceptible to amebic infection. The mechanism of *CREM*-mediated protection is under investigation. *CREM* might protect by conferring on epithelial cells resistance to amebic-induced apoptosis since *CREM* knockout mice had significantly higher caspase-3-positive cells upon amebic infection [39]. *CREM* could also confer protection by regulating the type-1 and type-3 cytokines (IFN-γ, IL-17A). IFN-γ and IL-17A protect against amebic infection, and it was observed that *CREM*-deficient mice had compromised differentiation of T helper (Th)1 and Th17 cells [13,40,41].

## A Healthy Microbiota Cooperates with the Immune Compartment to Shape the Protective Response

Different gut bacteria have been associated with both protection and disease progression in amebiasis. Verma *et al*., using qPCR, showed that *E. histolytica*-positive stool samples have a substantially lower abundance of *Bacteroides, Eubacterium, Clostridium leptum* subgroup, *Clostridium coccoides* subgroup, *Lactobacillus*, and *Campylobacter*, and a significantly higher abundance of *Bifidobacterium* [42]. However, the direct role of any of these bacterial genera in amebiasis was not investigated. *E. histolytica* is a colonic parasite and relies on colonic bacteria for survival and pathogenicity. One of the toxic factors that an ameba needs to adapt to in the colonic environment is oxidative stress. Several studies have revealed that enteric bacteria help amebae to resist oxidative stress [43-45]. Varet *et al*. recently identified that incubation with live *Escherichia coli* O55 increased *E. histolytica* resistance to H_2_O_2_-mediated killing by up to three to five times [44]. Interestingly, preincubation with *E. coli* O55 reversed the expression profile of more than 1000 genes in trophozoites that were observed to be modulated by H_2_O_2_ treatment [44]. *E. coli* malate dehydrogenase (MDH) is necessary for protection as treatment with MDH mutant *E. coli* could not protect from H_2_O_2_-mediated oxidative stress [43]. The addition of oxaloacetate to the medium, instead of *E. coli*, restored the protection. Overexpression of MDH in amebae could not maintain the defense, suggesting that bacterial MDH and its product oxaloacetate regulate the defense to H_2_O_2_-mediated oxidative killing. In addition to a survival advantage, the presence of particular bacterial species could be predictive of the symptomatic diseases. Gilchrist *et al*. analyzed *E. histolytica*-positive diarrheal stools and asymptomatic monthly stools collected from Bangladeshi children and showed that diarrheal stools have a significantly higher burden of *Prevotella copri* [15]. A similar observation was reported in a cohort of South African children [14]. This association requires further exploration to determine if *P. copri* is merely taking advantage of the inflamed gut or if it directly increases amebic virulence.

Decreased microbial diversity is a risk factor for amebiasis. Children with symptomatic *E. histolytica* infections had a decreased **Shannon diversity index** of the stool microbiota compared with children with asymptomatic infection [12]. To identify the mechanism of microbiota-mediated protection from amebiasis, our group recently developed a dysbiotic mouse model [12]. Mice were treated with an antibiotic cocktail in their drinking water for 2 weeks before challenge with amebic trophozoites. Upon amebic challenge, antibiotic-treated mice had significantly increased weight loss, clinical score, and trophozoite load in cecal contents. Remarkably, dysbiotic mice had fewer goblet cells with significantly decreased *Muc2* mRNA expression in cecal tissue. In agreement with these findings, other groups recently demonstrated the importance of gut microbiota in regulating the MUC2 mucin [46,47]. One of the potential mechanisms of protection is the regulation of immune cell recruitment at the site of infection. Antibiotic-treated mice had decreased CXC chemokine receptor 2 (CXCR2) expression and decreased recruitment of neutrophils in cecal tissue [12].

**Figure 2.**
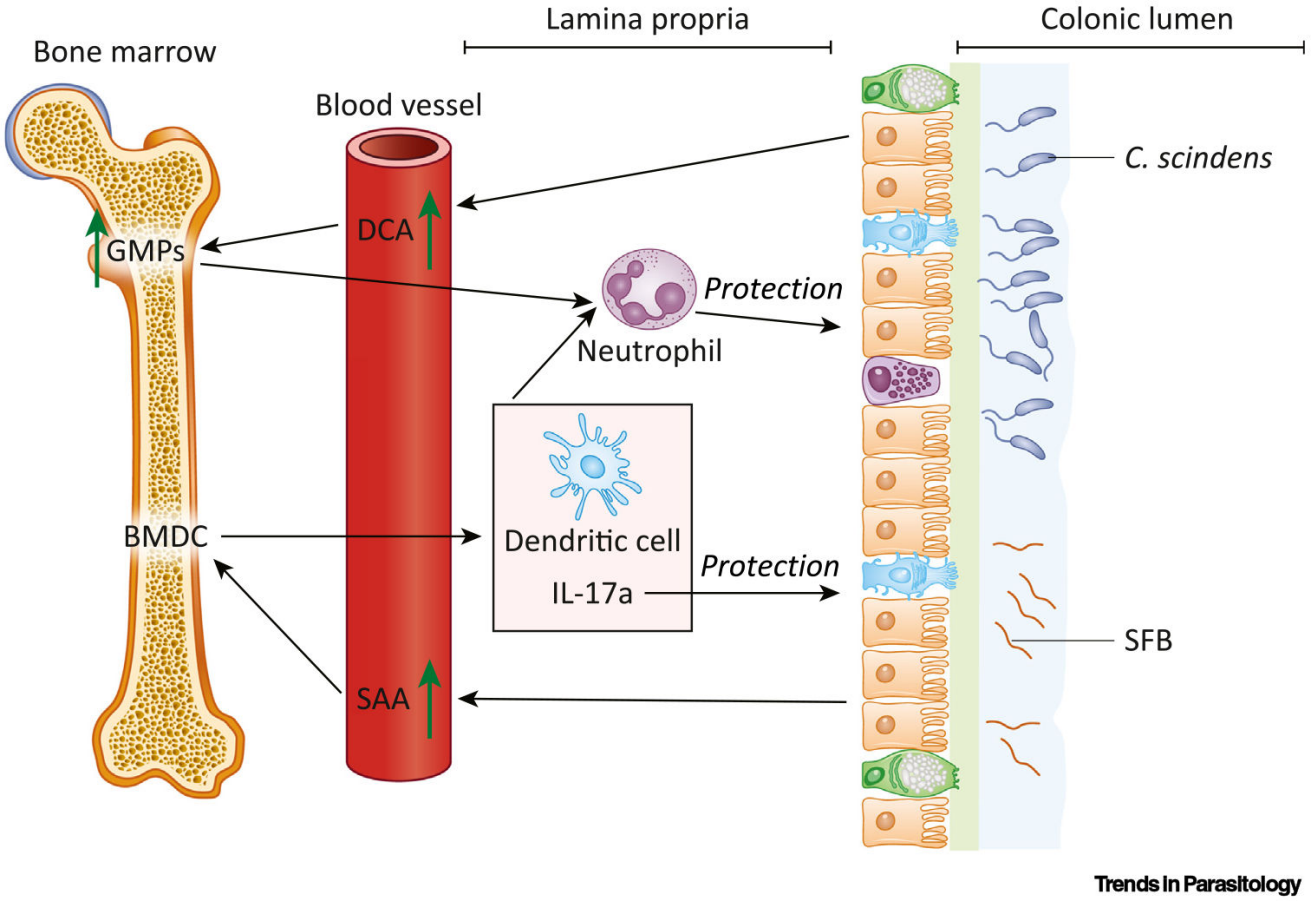
Microbiota–Bone Marrow Communication Mediates Immune Protection against Amebiasis *Clostridium scindens*' metabolism of cholic acid to deoxycholic acid (DCA) was sufficient to upregulate the generation of granulocyte-monocyte progenitors (GMPs) in the bone marrow. Increased bone marrow GMPs resulted in a higher level of colonic neutrophils following infection, resulting in protection from amebic colitis. Another mouse commensal segmented filamentous bacterium (SFB) interacted with bone marrow dendritic cells (BMDCs) through serum amyloid A (SAA). The SAA stimulated BMDC-orchestrated upregulation of colonic neutrophils, dendritic cells, and interleukin (IL)-17A during defense against amebiasis.

The microbiota–bone marrow axis is a relatively new area of study that scientists are exploring. Through modulating systemic responses, the microbiome can interact with bone-marrow stem cells to regulate the immune compartment [48]. Colonization with segmented filamentous bacteria (SFB) protected mice from amebiasis with an induced level of colonic IL-17A, IL-23, neutrophils, and dendritic cells and serum amyloid A (SAA) (Figure 2) [13]. Adoptive transfer of bone marrow dendritic cells (BMDCs) from SFB^+^ mice to SFB^−^ mice successfully transferred the protection in an IL-17A-dependent manner [13]. Burgess *et al*. recently discovered that intestinal colonization with *Clostridium scindens* is associated with increased bone marrow **granulocyte-monocyte progenitors** (**GMPs**) in specific-pathogen-free and gnotobiotic mice. Adoptive transfer of bone marrow cells from *C. scindens*-colonized mice to naïve mice conferred protection from amebiasis with an increased level of intestinal neutrophils and serum deoxycholic acid (DCA) (Figure 2). Importantly, treatment with exogenous DCA upregulated GMPs and protected from amebiasis [16]. These data clearly show the role of commensal microbiota in the regulation of bone marrow cells to maintain protection from amebiasis.

## Cell-Mediated Innate Immunity

### Neutrophils

Neutrophils can exert a defensive action to pathogenic infections by several mechanisms, including degranulation of the toxic factors, phagocytosis, and formation of neutrophil extracellular traps (NETs) [49]. Although there is some controversy, most of the studies have shown that neutrophils play a protective role during amebiasis [12,37,50,51]. Preventing neutrophil recruitment to the gut by anti-CXCR2 treatment or depletion of neutrophils by anti-Ly6G monoclonal antibody increased susceptibility to amebic infection in mice [12,37]. *In vitro* studies showed that neutrophils primed with IFN-γ, TNF-α, or lipopolysaccharide (LPS) have amebicidal potential [51]. The exact mechanism of how neutrophils might clear the amebic infection is not fully understood. Exposure of human neutrophils to live trophozoites, but not fixed trophozoites, induced the release of NETs in a time- and dose-dependent manner [52,53]. Interestingly, *E. histolytica* purified lipopeptidophosphoglycan was able to induce NET formation in a dose-dependent manner. It is not clear how amebic lipopeptidophosphoglycan induced NET formation while fixed amebae did not.

### Macrophages

Macrophages are one of the early responders to infections. By releasing inflammatory cytokines, such as IL-1β, TNF-α, and IFN-γ, and toxic nitric oxide (NO), macrophages can help to recruit other immune cells as well as exert direct amebicidal functions (Figure 1) [54-56]. Macrophages stimulated with colony stimulating factor (CSF), IFN-γ, or TNF-α are more potent in killing amebic trophozoites [57]. Treatment with NO downregulated the amebic virulence genes encoding cysteine proteinases and alcohol dehydrogenase 2 [58]. A recent study discovered that the interaction of trophozoites with human monocyte-derived macrophages and mouse bone-marrow-derived macrophages could induce the rapid secretion of the nuclear alarmin high-mobility group box 1 (HMGB1) [54]. Induction of HMGB1 might be the potential mechanism of the *E. histolytica*-mediated inflammatory response of macrophages since neutralization of HMGB1 dropped the expression of IL-1β and TNF-α [54,55,59]. Amebic attachment to macrophages induces **inflammasome** activation. *In vitro* experiments showed that culture of macrophages with trophozoites, but not the amebic lysate, produced activated caspase-1 and IL-1β within 10 min. Inhibition of NLRP3 inflammasome activation blocked caspase-1 and IL-1β activation [60]. Upon attachment, *E. histolytica* mediated cytoskeletal breakage of macrophages through a caspase-6-dependent mechanism [55].

Macrophage activation upon exposure to pathogens is a complex and context-dependent process. Classically activated or M1 macrophages are involved mainly in the induction of inflammation and killing of pathogens, while alternatively activated M2 macrophages are involved in tissue-repair mechanisms [61-63]. It would be worth exploring further to delineate the role of differentially activated macrophages in protection against amebiasis.

### Eosinophils

Eosinophilia is known to be associated with protection from bacterial and parasitic infections and disease progression in allergic diseases [64-67]. *Toxocara canis* antigen-induced eosinophilia protected gerbils from an amebic liver abscess [64]. However, this is not direct evidence of eosinophil-mediated protection since injection of *T. canis* antigen could have multifaceted impacts on the immune response [65]. Noor *et al*. recently discovered that treatment with recombinant IL-25 protected mice from amebiasis (Figure 1). This protection is thought to be conferred by IL-25-mediated eosinophilia since depletion of eosinophilia with anti-SiglecF administration abrogated the protection [17]. It is not clear if the ablation of basal level eosinophils can make mice susceptible to amebic infection. The downstream mechanisms of eosinophil-mediated protection from amebiasis are yet to be elucidated. The potential mechanisms could be: (i) regulating the production of mucosal secretory IgA, (ii) degranulation of toxic contents, (iii) secretion of cytokines, or (iv) governing production of AMPs [68].

In addition to neutrophils and macrophages, the recruitment of inflammatory monocytes was reported during amebiasis. In hepatic amebiasis, Ly6C^hi^ monocytes were involved in increasing the abscess volume [69]. Androgen-driven recruitment of Ly6C^hi^ monocytes was uncovered as a possible reason for sexual dimorphisms in disease pathology [70].

## Adaptive Immune Response

Immunization with several *E. histolytica* antigens, including the serine-rich *E. histolytica* protein (SREHP), Gal/GalNAc lectin, 29 kDa alkyl hydroperoxide reductase, a tetramer derivative of an anti-inflammatory pentapeptide, and heparan sulfate binding proteins have been shown to produce protective immune responses to amebiasis in animal models [71-76]. Roncolato *et al*. has recently shown that vaccination with the amebic surface metalloprotease protected hamsters from liver abscess [77]. There is also evidence in humans that an acquired immune response is protective: Haque *et al*. followed 202 Bangladeshi children from birth to 4 years of age and observed that the protection of children from subsequent *E. histolytica* infection was associated with the presence of anti-lectin stool IgA [5].

Mice vaccinated with a 64 kDa recombinant fragment of lectin (LecA), combined with the adjuvant alum, were protected from amebic infection and intestinal colitis [78,79]. Supporting the human data, LecA stimulated a robust mucosal IgA response in mice. However, mucosal IgA was not critical for the vaccine-mediated protection in mice since protection could be transferred to naïve mice by transferring CD3, CD4, or CD8 T cells. Besides a mucosal IgA response, immunization stimulated a robust IFN-γ and IL-17A response. Using different adjuvants with LecA gave partially different antibody responses; however, in all of them, IFN-γ was common and robust. Vaccinated mice lost protection to amebiasis when IFN-γ and IL-17A were neutralized [78,79]. Abhyankar *et al*. further improved the vaccination by adding liposome-based adjuvants in the formulation. Adding synthetic Toll-like receptor (TLR)-4 ligand and TLR7/8 ligand as adjuvant and all-*trans* retinoic acid as a coadjuvant further increased the mucosal IgA, IFN-γ, and IL-17A responses [18,19]. Interestingly, vaccination also induced a Th2 response (IL-4, and IgG1), which was previously thought to help in maintaining persistent amebic infection [41,80]. Effort should be continued to expound the role of the type 2 immune response in amebic infection. Considering the significant advances in the mouse model, a controlled preclinical and clinical trial of the vaccine is warranted.

## Concluding Remarks

Of the reported amebic cases, around 10–25% develop colitis, and 1% develop a liver abscess [81]. It is an enigma to scientists why one individual develops disease following colonization by *E. histolytica* while another does not. Further work studying host-protective factors will enable us to better understand how these variable outcomes occur.

Host defense to amebiasis is maintained by a composite interplay between microbiota, the epithelial layer, and the immune compartment. Host genetics, nutritional status, and other environmental factors further modulate this multilayered interaction. Contemporary research done by several groups has disentangled various aspects of the host response; that begins our understanding of the inconsistent outcomes of infection. Nevertheless, gaps remained to be filled in order to obtain a well defined picture of the host-protective immune response (see Outstanding Questions).

## Supplementary Material

1

## Box 1. Mechanisms of Cell Killing and Tissue Invasion

Ralston *et al*. reported a novel method of host cell ingestion and killing by amebae termed 'trogocytosis'. Following adherence of an ameba to a target cell, the parasite starts nibbling a part of the target cell and, over time, ingests multiple bites that eventually lead to cell killing [4]. The mechanism by which nibbling fragments of the target cell induces killing is not fully understood. Trogocytosis may break the membrane integrity of the target cells. Alternatively, the ameba might also inject acidic lysosomal content during nibbling, which may eventually cause cell death – as inhibiting the lysosomal acidification using weak bases inhibits trogocytosis [82]. However, this mechanism might not be unique to trogocytosis. Treatment with weak bases also impedes phagocytosis. Interestingly, blocking the amebic cysteine proteases, using the inhibitor E-64d, downregulated trogocytosis but not phagocytosis [83]. Although trogocytosis is one of the cell-killing mechanisms *in vitro*, the contribution of trogocytosis-mediated host cell death *in vivo* is unknown.

Intracellular calcium signaling in the ameba may also be involved in host cell killing. Pretreatment of an ameba with calcium channel blockers, or calcium chelators, significantly reduced the adherence and killing of host cells *in vitro* [84]. Increased calcium in the host cell induced by the ameba precedes caspase-3-dependent apoptosis. Inhibition of caspase, using pan-caspase inhibitor ZVAD, decreased amebic infection in mice [85]. At the end of the apoptosis, dead cells are phagocytosed by the ameba. Phagocytosis might help amebae to control excessive inflammation that could be cytotoxic for them. However, the role of phagocytosis in maintaining persistent infection has yet to be definitively determined [86]. A significant amount of work has been done to understand amebic phagocytosis. Recent work showed that amebic ESCRT (endosomal sorting complex required for transport) proteins and EhP3 (a homolog of the 14-3-3 family of proteins) are involved in phagocytic cup formation and actin reorganization during phagocytosis [87,88].

## Box 2. Mouse Model and Current Challenges

Finding an animal model that mimics human-like amebiasis was a limiting factor in the study of host defense mechanisms. Houpt and colleagues challenged C3H, CBA, C57BL/6, and BALB/c mice with log-phase trophozoites through intracecal laparotomy. While CBA and C3H strains remain infected for up to several weeks after the challenge – with colitis evidenced by immune infiltration, epithelial ulceration, and submucosal edema – the C57BL/6 and BALB/c strains were resistant to amebic infection [41,89]. Discoveries from the mouse model include the findings that deletion of *CREM*, the leptin receptor, and CD74, each individually increased susceptibility to infection [36,39,90]. It is important to mention here that children with polymorphisms in the leptin receptor and the *CREM* gene were found to have more symptomatic amebic infections [35,39].

Resistance to amebiasis in WT C57BL/6 mice might be orchestrated by innate signaling in the epithelial layer or due to a difference in the structure or function of the indigenous microbiota. The role of hematopoietic cells was ruled out as adoptive transfer of bone marrow cells from CBA mice did not make C57BL/6 mice susceptible [91]. Hematopoietic cells, while not responsible for the difference in resistance between CBA and C57Bl/6 mice, are important in defense against amebiasis. For example, IL-10 knockout C57BL/6 mice are susceptible to amebiasis. Transferring the IL-10-sufficient bone marrow cells to IL-10-deficient mice was adequate to protect them from amebiasis [91]. These data suggest that hematopoietic IL-10 is critical for the protection of C57BL/6 mice. C57BL/6 resistance to amebic colitis has been a challenge in amebiasis research as most of the genetically engineered mouse models have a C57BL/6 background. However, for specific questions, this limitation may be overcome by using double-knockout mice generated by crossing a mouse deficient in a gene of interest with IL-10 knockout (KO) mice. In addition to IL-10 KO mice, C57BL/6 RAG1 KO mice are also sensitive to amebiasis [16]. This observation is interesting because it suggests that adaptive immunity may play a critical role in early protection from amebiasis indirectly via the altered microbiota of RAG1 mice [92]. Cohousing WT with RAG1 mice might make WT mice susceptible to amebiasis. In addition, antibiotic-induced dysbiosis renders C57Bl/6 mice susceptible to amebiasis [12]. These approaches may help researchers to utilize genetic models without resorting to losing a key immune pathway (as one would using an IL-10 KO mouse) to study host pathways that affect amebic infection.

## Outstanding Questions

Does MUC2 protect from long-term colonization of trophozoites in the gut?

Are other members of the mucin family (MUC1 or MUC6) important in protection from tissue damage and *E. histolytica* infection? Do membrane-bound mucin and gel-forming mucin contribute differently in conferring protection?

What is the role of AMPs *in vivo*? Are AMPs alone sufficient to confer *in vivo* protection from amebiasis?

Are group 2 innate lymphoid cells (ILC2) involved in IL-25-mediated protection from amebiasis?

How do amebae interact with tuft cells to downregulate the secretion of IL-25?

What are the downstream mechanisms of eosinophils during IL-25-mediated protection?

What is the contribution of classically activated macrophages and alternatively activated macrophages in protection from amebic colitis?

How do neutrophils exert their amebicidal functions?

In addition to regulating neutrophil recruitment to the site of infection, does the gut microbiota modulate neutrophil effector functions?

Where does CREM act (on immune cells or epithelial cells) to protect from amebiasis?

Does a type 2 immune response play a role in vaccine-mediated protection?

## Highlights

Vesicle-mediated exocytosis of MUC2 is critical in providing protection against tissue damage, and MUC2 regulates antimicrobial peptide production by Paneth cells during *Entamoeba histolytica* infection.

While certain pathobionts can defend the ameba from oxidative stress in the colon, other commensal microbiota can protect the host by controlling the recruitment of neutrophils.

Amebic infection downregulates interleukin (IL)-25 production in mice and humans. Exogenous treatment with IL-25 induces eosinophilia to confer protection.

Children with a polymorphism in transcription factor cAMP response element modulator (CREM), and *CREM* knockout mice, are more susceptible to amebiasis.

Vaccination with amebic surface protein LecA combined with Toll-like receptor (TLR)4 and TLR7/8 agonists induces a robust mucosal IgA, interferon (IFN)-γ, and IL-17a response to provide protection from amebiasis.

## Glossary

Adjuvant: a synthetic or a biological molecule that is added to vaccines to induce a robust and long-lasting immune response. Quite often, adjuvants are designed to activate the inflammasome or a pattern-recognition receptor such as a TLR.
Amebic cyst: amebae undergo encystation by forming a double-layered wall before exiting the host. Inside the cyst, amebae are immotile and metabolically inactive. This amebic cyst enables an ameba to survive for up to several weeks outside the host.
Antimicrobial peptides (AMPs): peptide molecules that are cytotoxic to microbes and are released as a part of host defense. In the intestine, AMPs are mainly released from Paneth cells.
Cysteine protease: an amebic virulence protein involved in cleaving the host mucus layer and binding amebic trophozoites to host cell membranes.
Gal/GalNac lectin: an amebic surface protein necessary for virulence; it can bind host cell membrane carbohydrates d-galactose or *N*-acetyl-d-galactosamine.
Genome-wide association study: survey of a genome by mapping SNPs to test for genetic variation among individuals associated with a disease or particular trait.
Granulocyte-monocyte progenitors (GMPs): bone marrow-derived progenitor cells that can give rise to neutrophils, monocytes, and dendritic cells.
Inflammasome: a multiprotein complex inside the cell that activates IL-1β, IL-18, and gasdermin to initiate inflammation.
Pathogen-associated molecular patterns (PAMPs): microbial molecules, such as endotoxin or peptidoglycan, that are recognized as foreign by host cell pattern-recognition receptors such as Toll-like receptors (TLRs) to initiate inflammation.
Shannon diversity index: a mathematical measure of the diversity of a bacterial community, for example, within the gut microbiome. This index takes into account both the abundance and the evenness of the species within a community.
SNP: a change in a nucleotide at a specific site in the genome that is found in a fraction of the population. Although most SNPs are not known to have any functional impact on the genome, some have been found to be associated with, and in some cases to be the cause of, susceptibility to infectious diseases.
